# A process-based framework to guide nurse practitioners integration into primary healthcare teams: results from a logic analysis

**DOI:** 10.1186/s12913-015-0731-5

**Published:** 2015-02-27

**Authors:** Damien Contandriopoulos, Astrid Brousselle, Carl-Ardy Dubois, Mélanie Perroux, Marie-Dominique Beaulieu, Isabelle Brault, Kelley Kilpatrick, Danielle D’Amour, Esther Sansgter-Gormley

**Affiliations:** 1 Faculty of Nursing, University of Montreal, C.P. 6128 succ. Centre-Ville, Montréal, Québec H3C 3J7 Canada; 2 Department of Community Health Sciences, University of Sherbrooke, 150, place Charles-Le Moyne, Bureau 200, Longueuil, Québec J4K 0A8 Canada; 3 Department of Family Medicine and Emergency Medicine, University of Montreal, Pavillon Roger-Gaudry, 2900, boul Édouard Montpetit, Montréal, Québec H3T 1J4 Canada; 4 School of Nursing, University of Victoria, PO Box 1700 STN CSC, Victoria, BC V8W 2Y2 Canada

**Keywords:** Collaboration, Delivery of health care, Integrating process, Logic evaluation, Nurse practitioners, Practice model, Primary health care, Role definition, Team support

## Abstract

**Background:**

Integrating Nurse Practitioners into primary care teams is a process that involves significant challenges. To be successful, nurse practitioner integration into primary care teams requires, among other things, a redefinition of professional boundaries, in particular those of medicine and nursing, a coherent model of inter- and intra- professional collaboration, and team-based work processes that make the best use of the subsidiarity principle. There have been numerous studies on nurse practitioner integration, and the literature provides a comprehensive list of barriers to, and facilitators of, integration. However, this literature is much less prolific in discussing the operational level implications of those barriers and facilitators and in offering practical recommendations.

**Methods:**

In the context of a large-scale research project on the introduction of nurse practitioners in Quebec (Canada) we relied on a logic-analysis approach based, on the one hand on a realist review of the literature and, on the other hand, on qualitative case-studies in 6 primary healthcare teams in rural and urban area of Quebec.

**Results:**

Five core themes that need to be taken into account when integrating nurse practitioners into primary care teams were identified. Those themes are: planning, role definition, practice model, collaboration, and team support.

The present paper has two objectives: to present the methods used to develop the themes, and to discuss an integrative model of nurse practitioner integration support centered around these themes.

**Conclusion:**

It concludes with a discussion of how this framework contributes to existing knowledge and some ideas for future avenues of study.

## Background

Major challenges for developed countries’ health systems in the next decades include pervasive health inequalities; limitations in health services accessibility, care comprehensiveness, and continuity, especially in primary care; demographic shifts; technological developments; and fiscal constraints [1-7]. The pressing nature of these challenges should not, however, obscure the fact that, at the programmatic level, there is strong evidence on effective intervention paths. Among those, increased reliance on primary care over specialized hospital-based care and a greater role for nurses and other non-physician professionals in primary care teams are of particular importance to simultaneously improve efficiency and accessibility [8-12]. In this general context, the current article is focused on one specific objective, which is to provide evidence-based, practical advice to support the effective integration of primary care nurse practitioners (NP) into care delivery systems. We have pursued this objective using an original research strategy combining results from logic and implementation analyses [13].

There is a large body of evidence suggesting that increased reliance on NPs has the potential to improve accessibility of primary care services while controlling expenditures [8,14-20]. However, integrating NPs into primary care teams has proven challenging in practice [8,19,21,22]. There is abundant literature analyzing the underlying causes of those challenges, but operational literature on the solutions to overcome them is considerably more limited.

### Context

In 2010, Quebec’s government announced it would support NP practice and fund the integration of 500 primary care NPs over the next decade. The main objective put forward was to improve accessibility [23]. This decision was the starting point for a large-scale research project focused on supporting primary care teams that integrated NPs as they went through the process of rethinking care delivery models, processes, and roles.

The majority of healthcare services in Quebec are funded through a Beveridgean public insurance system. Essential Care, whether offered in publicly owned institutions or in private medical clinics, is usually free at the point of service. The Ministry of Health and Social Services (MSSS) funds services through public taxation and has a direct responsibility in the overall governance of the healthcare delivery system. When the government decided to add 500 NPs to the healthcare system, the MSSS had a central role in drafting, implementing, and supervising a “deployment plan” that would reach the broader policy objective of improving accessibility to primary care services. For example, students in NP master’s programs are offered a generous bursary package by the MSSS to support their studies and professional travel expenses in exchange for a commitment to work at least three years in a location approved by the Ministry.

In Quebec, primary care NPs are registered nurses who have successfully completed a master’s-level, university-based, NP program. Upon employment they are required to work in collaboration with at least one physician, with whom they sign a “partnership agreement”. NPs have the legal and regulatory authority, in collaboration with a physician, to assess, diagnose and treat patients for acute common illnesses and injuries, manage chronic diseases, provide pregnancy care up to 32 weeks of gestation, and engage in health and wellness promotion. They order and interpret diagnostic tests, prescribe drugs (based on a formulary) and perform specific procedures within their legislated scope of practice [24]. Upon completion of their educational programs, and prior to registration, NP graduates are accorded the right to practice under medical supervision as “candidates” and have two years to pass the certification exam jointly drafted by the nursing and medical professional boards.

NPs are expected to provide primary care in public organizations providing primary care and social services (CLSCs); hospital-based family medicine units (UMFs), which train medical residents in family medicine; and family medicine groups (GMFs). GMFs are private medical clinics where public hospitals cover the salary and benefits of nursing staff (both RNs and NPs) in exchange for clinics providing extended opening hours and increased care continuity.

## Methods

**Figure 1:**
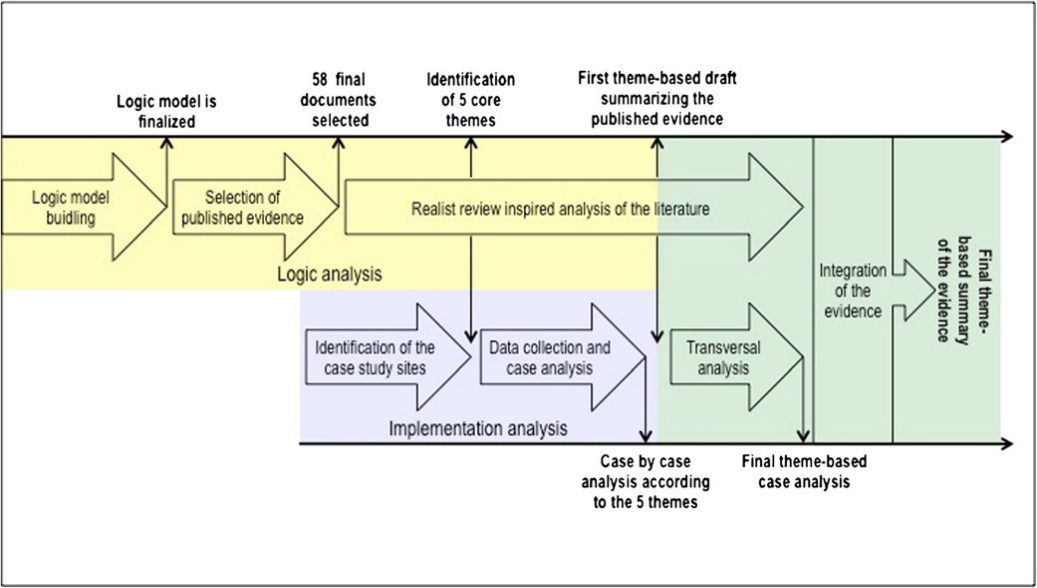
**Research process.**

In this paper we report on finding obtained from an original research strategy combining results from logic and implementation analyses [13]. The logic data were derived from published literature and implementation data were derived from case studies conducted by the research team in Quebec (Canada). The recommendations presented here are based on a combined logic and implementation analysis. Both evaluation approaches aim to assess the potential value of a given intervention, but each has a different focus. On one hand, “*Logic analysis is an evaluation that allows us to test the plausibility of a program’s theory using available scientific knowledge—either scientific evidence or expert knowledge*” [25]. Implementation analysis, on the other hand, relies mostly on empirical observations to identify factors that actually enhance or impede the implementation of the intervention or the production of its effects [26]. Combining these two evaluation approaches allowed us to build a comprehensive understanding of factors and contextual characteristics potentially influencing NP implementation, which in turn made it possible to provide evidence-based advice to optimize implementation and maximize NPs’ effectiveness. Furthermore, implementation analysis was helpful in identifying which determinants of implementation effectiveness were more important than others as deployment of NPs was being planned and phased-in. At the operational level, the research team first conducted a logic analysis of Quebec’s NP deployment plan and of NP practice patterns, mostly based on a realist review of the literature and on expert advice. We then conducted an implementation analysis using a case study research design (n = 6 cases) in three health regions of Quebec. The evidence derived from both the logic and implementation analyses was then combined into practical advice pertaining to five core themes that structure the NP integration process. Figure 1 below represents phases of the research process schematically.

### Logic analysis

First we began with a logic analysis which is a three-step approach [25,27] consisting of building a logic model, developing the conceptual framework, and evaluating program theory. To build the logic model, we consulted available documents produced by the Ministry, regional boards, professional organizations and experts from the MSSS who were directly involved in the NP implementation plan in Quebec. We then reviewed the available published evidence through a method inspired by the realist review approach [28-31]. Using the logic model of the MSSS’ NP implementation plan we established as a starting point, we iteratively built, from the literature, a conceptual model of the best practices and supporting conditions for NP integration into primary care teams.

Given the complexity of NP implementation, purely keyword-based search syntaxes were unlikely to provide satisfactory results [28,31-34]. As a starting point, we chose instead to conduct a manual search in the Advanced-Practice Nursing (APN) Literature Database [35], in which all the scientific literature published on advanced practice nursing between 2000 and 2009 had been systematically compiled. We extracted the 3,674 references identified as relevant to advanced-practice nursing in the APN database and manually assessed their relevance for the purpose of our study based on titles and abstracts. A total of 159 articles were retained for further review. To be retained, documents had to address NP implementation in primary healthcare teams, practice models, or integration processes. Two members of the research team independently assessed the retained articles for relevance.

Next documents were summarized using an abstraction tool and given a relevance score and a scientific validity score, both ranging from 1 to 3. The relevance score ranged from 1 for documents offering a minor or marginal contribution to the understanding of the phenomena studied to 3 for documents providing detailed insights directly focused on those phenomena. The validity score ranged from 1 for an editorial opinion or significantly flawed research to 3 for an article presenting results from a robust and well-conducted method. For editorial type material with no discernable evidence base, reviewers also had the option of removing the article from the database (score of 0). Only documents with a combined score (relevance and validity) of 4 or higher, 43 articles were retained as primary sources for analysis.

At the time of conducting the review, the APN Literature Database was limited to literature published between 2000 and 2009. To include publications after 2009, we reproduced the search syntax used to compile the APN Literature Database to identify articles published between 2010 and 2012. We applied the same sorting methods to this second corpus of articles and retained 53 articles for full textual analysis. Of these, 15 were added to the 43 documents selected in the first phase. Altogether 58 documents were selected for in-depth analysis.

The documents, both peer-reviewed articles and research reports, were then iteratively read, often several times, and analyzed to build a preliminary conceptual model according to the realist review approach [28,31,34,36]. The model was focused on structuring available evidence to support NP integration. From the literature, five major themes were inductively identified as the conceptual model’s core elements: 1) planning the integration, 2) role definition, 3) patient management, 4) collaboration, and 5) support to the team. For each theme, we produced a first summary of the information collected in the literature review. At this point, the interdisciplinary expertise of the research team (which included registered nurses, NPs, physicians, and experts in organizational theory and health administration) was applied to identify new documents on an ad hoc basis. The draft summary for each theme was then used as the analytical framework for the implementation analysis.

### Implementation analysis

The second data source, upon which the advice provided here is based, comes from an implementation analysis using six qualitative case studies. Each case was defined as a clinical team into which one or more NPs had been integrated in Quebec. We identified potential cases in collaboration with MSSS and with the Regional Health and Social Services Agencies (ASSS) involved. Cases were selected based on two criteria. First, cases had to involve teams whose NP integration was seen as successful by the MSSS or the ASSS, in order to identify and analyze successful integration models. Second, to improve the external validity of the findings [37-39] we deliberately sought maximum variation in terms of environment (rural, suburban, urban), organizational setting (privately owned clinics, community-based publicly owned clinics, and hospital-based primary care teams), and stage of NP integration. Table 1 below provides additional information on the characteristics of each case study site.

**Table 1:** **Location and team composition for each case study site**

	Case 1	Case 2	Case 3	Case 4	Case 5	Case 6
Location	Urban	Rural	Rural	Rural	Urban	Urban
**Type and number of professionals within the team**	2 NPs, 67 MDs, RNs, nursing assistant, social worker, psychologist, nutritionist, kinesiologist	1 NP, 15 MDs, 2 RNs, 1 nursing assistant, 1 nutritionist	2 NPs, 2 MDs, 4 RNs, 2 nursing assistants, 3 social workers, 1 occupational therapist	2 NPs, 2 MDs, 1 RN, 1 nursing assistant	1 NP, MDs and residents, RN, nursing assistant, pharmacist	1 NP, 3 MDs, RNs, social worker, occupational therapist, physiotherapy technicians, pharmacists

Findings from the case studies are based on 34 semi-structured interviews conducted with members of the clinical teams and other key actors as well as on analysis of available documentation. In each setting, researchers skilled in interviewing conducted interviews with the main stakeholders involved in the NP integration into the primary care team, such as the NP, the physician partner, and the Chief Nursing Officer of the local hospital, and most of the nurses and the administrative staff of each of the primary care teams. All informants gave informed consent, and best practices for the ethical conduct of research were followed. The project was approved and supervised by the research ethics committees of all institutions involved. Each of the six cases was under the responsibility of one team member, who produced a narrative case summary per case, structured around the five themes identified in the literature review. Those summaries were discussed in research team meetings, and cross-case insights were identified. In a second step, we produced five narrative theme-based cross-analyses of the cases to synthesize the contribution of empirical case-study evidence according to each of the five previously identified themes.

### Combined analysis

After completing the implementation analysis, we revised the summaries constructed during the logic analysis phase to incorporate empirical knowledge derived from the case studies. Ultimately, the evidence from the logic analysis and the implementation analysis was integrated into one single theme-based narrative, and the specific expertise of each team member was mobilized in that process. The objectives of using two different approaches to analysis were: 1) to be able to cross-validate and compare the evidence derived from the literature to practices identified in our case studies and; 2) to assess the applicability and usefulness of the literature-based advice in real-world contexts and determine which of the factors identified in the literature were most important in supporting the implementation of primary care NPs; and 3) to better appraise implementation dynamics and understand how factors are intertwined during the implementation process.

Three important points should be made regarding the final integration of the material. First, our focus was to offer practical advice. Much of the literature is structured around identifying barriers and facilitators [8,40-44] but offers little to support teams’ improvement. It might also be worth stressing here that integration is a process, and thus a dynamic phenomenon, whereas a list of barriers and facilitators is a very static analytical framework. Second, we aimed for a single set of theme-based recommendations for all team members, whether NPs, RNs, MDs, administrators, or support staff. What is your third point?

## Results

As described in the Methodology section above, unless otherwise specified, the findings provided here come from the integration of evidence from the logic analysis and implementation analysis components of the study and are structured around the five themes identified.

### Planning to integrate an NP: an opportunity for clinical teams

The first element in successfully integrating an NP into a primary care team is advance planning. Although this may seem obvious, our data suggest inadequate planning is all too common [21,22,45]. The first step in the planning process is to reflect on the intended practice model in discussions among all the different actors involved (physicians, nurses, managers, and other members of the team). A comprehensive plan developed collectively by all team members is a key factor in implementing an effective and satisfactory practice model. In practical terms, it is thus important to take into account the time and energy this process will require from the clinical team and the consequent timeline [46].

Our analysis suggests there are often preconceived notions about the nature of NPs’ training and practice [47]. It is also important not to underestimate the distance between making administrative and regulatory information about NPs’ role and scope of practice available and ensuring that all team members are aware of this information. Preparation is a matter not only of making information available but also of transforming available information into practical knowledge [48-50].

More broadly, the arrival of an NP should be seen as an opportunity to reflect on the current practice model’s strengths and weaknesses and to establish a shared vision of the desired future practice model. This reflection should cover certain fundamental considerations, such as fit between patients’ needs and appropriate response, solutions to improve the practice model and role of each professional in the team [21,51-55].

Once the practice model is defined, the broad dimensions of the NP’s role and expected contribution should be discussed. The NP’s actual role needs to be discussed during the hiring process and determined in collaboration with the NP hired; however, by defining broad dimensions beforehand, the team will be able to assess whether their expectations are realistic and consistent with the regulatory and administrative framework governing NP practice. A team-generated definition also provides a useful tool for candidate interviews. It will also be important to take into account the level of experience of the NP hired and the potential evolution of that person’s practice with growing experience and abilities. All available evidence suggests the first year of practice after graduation is one of transition [42,45,46,56,57].

Our data suggest that a key operational factor is to formally designate a person to be in charge of the practical steps of the integration process, including setting up communication strategies to ensure effective information transmission within and outside the team [45,58].

Finally, throughout the process, it is important to both conceive of and present the arrival of an NP as an opportunity for the whole team to improve by reflecting on how things are currently done, identifying areas for practice improvement, and creating a vision for the entire team’s future practice.

### Role definition and consensus building

The importance of appropriately and coherently defining the NP’s role and scope of practice is, by far, the point most often made in the literature on obstacles to collaboration or to NP integration [40,59-62]. The results from our combined analysis show that, even when all clinical team members share the desire to develop a collaborative practice, misunderstandings and conflicts around roles are frequent and significant barriers to NP integration and practice. When the NP’s role set is well-defined, there is consensus about how patient management responsibilities are distributed, each team member’s skills, and scope of practice, as well as differences and similarities of roles [63].

Overall, there is solid evidence to support the need for team consensus on role definition. However, evidence to support more instrumental recommendations on how to create such a consensus inside interdisciplinary care teams is much weaker. The optimal level of role formalization, in particular, is open to debate. The level of formalization describes the extent to which each person’s role is defined, in more or less detail, in written documents. Some [51,56] suggest that the role definition process should result in each person’s role being formalized in writing. However, we have found no strong empirical evidence to support the conclusion that role formalization is the sole or best way to support consensus around role definitions. One hypothesis derived from our study is that the optimal level of formalization is a function of team size and that larger teams may require greater formalization. In any case, role formalization should be sufficiently flexible and malleable to allow team members’ practices to evolve [64,65]. Excessive role formalization that attempts to set down in writing every possible situation and all interventions is probably counterproductive to collaboration [65,66].

Notwithstanding the level of formalization, a central element in the process of defining the NP’s role is, in fact, the recognition that the process cannot be limited to the NP’s role. Coherently defining the NP’s role and practical scope of practice involves rethinking everyone’s role z [53,67,68]). Failure to do so is likely to produce role overlaps, redundancies, and frustrations. Sibbald, Laurant and Scott [69] proposed a useful typology for defining primary care roles using four logics:

- enhancement, which involves widening the field of practice or the competencies of a professional group;
- substitution, which involves replacing one type of professional by another in the provision of certain services;
- delegation, which involves allowing a subordinate professional to provide extended services, but under the supervision of another type of professional;
- innovation, which involves establishing new types of services or creating new professional roles.

These logics are not mutually exclusive but can serve as guideposts for thinking about the process of redefining roles in a team. As a general rule, role definition should enable all team members to:

- practice to the full scope of their capacities;.
- contribute efficiently and effectively to patient management according to each professional’s expertise.
- develop their own expertise and capacities and facilitate this development process.

At the practical level, an essential factor in the role definition process is the identification of one or more project champions in the organization, such as chief nursing officers or nurse consultants, who will help ensure the full scope of the NP’s practice is respected. It is also helpful to repeat periodically the interactive process of discussing team members’ roles, as those evolve over time.

### Several patient care models but no simple recipe

The practice model for NPs in Quebec, as described in official documents and regulations [70-77], is one in which NPs and their physician partners look after patients’ needs collectively. In practice this general principle can take two different forms: the “joint model” and the “consultative model” [21,51]. A model is considered *joint* when the NP and the physician partner follow the same panel of patients. In such a model, both professionals may see the same patients at different points in their treatment. Conversely, a model is considered *consultative* when the NP and the physician partner each follow a different panel of patients and the physician is consulted as needed. In that model, most patients followed by the NP never see the physician except for the occasional specific need. Following the evidence derived from the literature, we used three dimensions to assess the suitability of patient care models: *group* practice, *interdisciplinary* practice, and *collaborative* practice [67,78-82]. *Group* practice is characterized by team members’ sharing of resources and responsibilities. In *interdisciplinary* practice, the patient management model is based on pooling the complementary expertise of the various professionals. Lastly, we describe as *collaborative* the communication and task-sharing processes that optimize efficiency and quality of care. A coherent definition of the practice model is a crucial determinant of the quality of interprofessional collaboration and of the capacity to establish operational definitions of each team member’s role. There is also credible evidence to suggest the coherent definition of a patient care model is an important determinant of job satisfaction in primary care interdisciplinary teams [83].

Three general observations emerged from our implementation analysis regarding the patient care models implemented. First, the models implemented by the teams were generally not the result of an explicit choice. They seemed rather to have emerged through trial and error. Second, our data show that, in practice, the models implemented were generally hybrids of the two types presented here. Several regulatory factors, such as procedures for enrolling patients with physicians or clinics, had a determining influence on the patient care models created. Lastly, in the cases analyzed, consultative management figured much more frequently than joint management. This could be the result of a better fit between the consultative management model and structural characteristics of Quebec’s healthcare system. It might also have to do with many primary care physicians’ limited experience of working collaboratively or from how physicians expect to practice with other physicians.

Overall our results do not allow us to suggest that one patient care model is inherently better than the others. It is likely that a model that works well in one setting may be inappropriate in another. On the other hand, there is convergent evidence to support the notion that it is the overall coherence of the model that matters [55]. If the patient management model is incompatible with the types of clientele followed, with the team’s composition and collaboration process, or with the NP’s level of experience, its operation will be both dysfunctional and frustrating [9,84]. Examples of dysfunctions observed included difficulties in assembling a sufficient patient panel for the NPs, or a non-functioning consultative model due to overly stringent interpretation of procedures for the NPs’ practice; such dysfunctions were symptoms that the models needed to be reviewed and adapted [47].

There appear to be three determining factors to consider in choosing a patient management model: clientele characteristics, physicians’ and NPs’ experience and preferences, and number of physician partners. The nature and complexity of the clientele followed must be consistent with legislative and regulatory frameworks for NPs’ scope of practice, including the range of diagnostic tests and drugs they can prescribe, and procedures for referring to specialists [85,86]. Teams that opt for a consultative patient management model need to establish parameters regarding the characteristics of patients followed by the NP so that the NP is able to meet most of those patients’ health needs [9]. It is also important to understand that this implies a potential increase in the average complexity of the patients followed by the physicians in the team. The data from our case studies suggest that physicians caring for more complex patients could impact the amount of time required for the patient visit and, in a fee-for-service scheme, physicians’ revenues.

As far as NP’s experience and preferences are concerned, there is convincing evidence that their first year of practice is one of transitioning toward fully occupying their scope of practice and developing autonomy. It is thus important that the patient management model be allowed to evolve over time as the NP gains experience and confidence. As well, somewhat akin to the great variability seen in general physicians’ practice profiles, the preferences and skills of both the NP and the physician partner should play a role in developing patient care models. Here again, it appears important to keep these parameters open and to be ready to redefine them over time.

The third element to consider is the number of different physician partners with whom the NP will need to collaborate [55]. While the literature is not specific on the optimal number of physician partners per NP, the difficulties encountered in our cases suggest that the optimal number of physician partners is probably between two and four. Having only one physician partner results in logistical challenges when that physician is absent. Conversely, the more physician partners there are, the more adaptation is required from the NP, as bonds of trust are built up slowly and differently from one person to another.

### Collaboration: a tool for optimal patient care

The fourth theme that needs to be taken into account by interprofessional primary care teams, whether they include NPs or not, is that of collaboration processes. This is the focus of a huge body of literature, which we will not try to summarize here. However, it is worth remembering that good collaborative relationships among professionals foster a positive work climate and help to optimize quality of care and patient management [87-89].

The extensive literature on collaboration suggests determinants can be organized into three levels: *interpersonal*, which includes elements such as confidence, attitudes, and communication skills; *organizational*, which encompasses leadership, egalitarian relationships, communication, coordination, and role clarification; and *systemic*, which refers to regulatory environments, funding, and remuneration, as well as educational frameworks [89,90]. At the practical level, three elements seem to stand out as particularly important. First, it is important to identify leaders, both managers and clinicians, to whom team members can turn for support to settle differences, resolve problems, or provide help in situations where communication is problematic [46]. Second, developing collaboration among clinicians, whether inter- or intraprofessional, requires time, in particular for mutual trust to develop. For this trust to be built, new NPs need to demonstrate their competence in managing patients. Although much of the literature focuses on relations between physicians and NPs, collaboration between NPs and nurse clinicians is also a key issue. Our empirical data show a period of adjustment is required during which nurses can get to know each other and talk about their visions and respective responsibilities, building up their collaborative relationship over time. Time also needs to be allocated to give team members opportunities to talk about values and their vision of the role and how it can contribute to service provision [21,46,63,67]. Space is also a strategic element in collaboration. Professionals need space in which to be able to meet and talk together both formally and informally.

Finally, collaborative practice does not always emerge spontaneously [21,88,91]. The literature on training for physician–NP collaboration identified in our review recommends a variety of learning strategies, such as case discussions, scenario building, and discussions around clinical and organizational issues [8,55,56,67,88-90,92,93]. Our implementation analysis data suggest that NPs greatly appreciated activities involving joint training or clinical case discussions and considered them to be team-building activities to construct a joint practice. Focusing discussions on quality of care and emphasizing a patient-centered approach are also good ways to foster productive team discussions.

### Supporting teams integrating an NP

Professionals’ capacity to develop effective and satisfactory clinical practices depends primarily on the energy, openness, and mutual trust of the clinicians themselves. Yet it is important not to underestimate the key roles of managers, nursing and medical directors, and administrative assistants in supporting practice and its development [58,64,94]. Our study identified three complementary spheres of activity needed to adequately support primary care teams integrating NPs: clinical-level support, team-level support, and leadership and systemic support.

#### Clinical-level support

The data from our implementation analysis coincide with findings from experiences in other provinces and countries showing that some NPs are not able to fully exploit their roles due to issues related to drug prescribing, diagnostic testing, and receiving consultation reports from specialist physicians [8,21,51,86,95,96]. These problems are sometimes caused by administrative failures and sometimes by the opposition of certain professionals. Support provided to NPs at both the clinical and systemic levels is essential to smooth out these difficulties [89,91,97].

NPs also need to be able to develop their clinical judgment and decisional autonomy and apply these in practice. These are competencies that develop over time and depend on the quality of interprofessional collaboration and the comprehensiveness of the patient care model [21,88,91,98,99]. Likewise, access to continuing education is an important factor in developing good clinical practice, not only for NPs, but also for physicians and other professionals [63]. The lack of availability of specific training, difficulties in being liberated from work, and distance to training locations were identified as significant obstacles in this regard.

In those settings where the NPs’ scope of practice was most extensive and where their role definition was evolving positively, several determining factors were observed: joint meetings among managers, nursing or medical directors, and partner physicians; a shared vision of the NP’s role; mobilization of the care team members’ complementary expertise; and collaborative work with nurse clinicians. The NPs’ prescribing authority and decisional autonomy were also discussed and clarified by the whole team, including the medical team.

#### Support for the team

There is solid evidence that strong leadership and consistent support to primary care teams foster the emergence of an effective patient management model [21,40,64]. Our cases showed great variability in the administrative structures in place and in the persons mobilized (e.g. clinic managers; head nurse; licensed practical nurses; physician clinic manager; manager, etc.). Only rarely were there clear lines of authority delineating the responsibilities of managers at different hierarchical levels. Organizational theory suggests that the characteristics of primary care teams (small professional groups, very autonomous participants, decentralized power in terms of operations) favor informal functioning and structures that are not very hierarchical [66,100]. This type of structure produces good results when there is a consensual vision of the organization’s goals and values but carries the inherent risk that no one would feel accountable for resolving problems. There thus needs to be positive leadership from one or more key persons who have strong legitimacy and a clear sense of purpose.

Effective communication mechanisms are a key factor in encouraging the emergence and maintenance of a shared vision of the team’s objectives and values [45,58]. Communication must be balanced between formal and informal opportunities for exchange. Similarly, a balance is required—depending on the persons, subject, and context involved—between direct communications (such as discussions between two professionals to improve a suboptimal work practice) and indirect communications (such as transmitting suggestions for improvement to the person in charge of a specific aspect) [56,58,90]. In any change process, it is normal that tensions and differences in preferences would arise between team members. In some cases, tensions are best resolved by face-to-face discussion. Resolving disagreements directly within the team is part of the process of creating team dynamics. Even so, it is essential to be able to consult a neutral third party when necessary, someone prepared to take on a boundary-spanning role between the medical and nursing disciplines. Here again, there is no solid evidence for any specific operationalization, but several credible data sources in the literature suggest the principle itself is important. This person who has the legitimacy, capacity, and motivation to take on this role will be responsible for preserving an overall vision of all the work processes [58].

#### Systemic support

Beyond their internal functioning, primary care teams are also part of larger healthcare systems. As such, the operations of primary care teams are also structured by the environments in which they practice, with regard to such things as billing policies, enrolment of new patients, or referrals for tests or specialized services. As with any other practice change, introducing an NP entails adjustments and communications between the primary care team and its external environment [85]. However, the NP role is still evolving, and part of that role is played out at the interface between medicine and nursing. To fulfill their responsibilities, NPs must be able to rely on collaboration from other actors in the external environment (specialist physicians, diagnostic services, pharmacists). It is therefore important that clinical teams be given the systemic support needed to identify appropriate solutions and to ensure problems are resolved [14,88,91]. Fulfilling this mandate takes time and a good knowledge of the local environment. This is why, in practice, the functions of direct supervision and systemic support may need to be shared among the local leaders [58].

Finally, while it is useful to divide the discussion on support for practice into three spheres here, it is also important to remember they are interdependent and, in practice, necessarily integrated.

## Discussion and conclusion

The results of our logic and implementation analyses suggest the existing literature on NP integration could be improved in two ways. First, taken as a whole it is too often intradisciplinary, offering analysis and advice that is too targeted to one professional group. Yet, by definition, integrating NPs into primary care teams is an interprofessional endeavor. Second, while the literature offers much converging descriptive evidence regarding barriers to and facilitators of NP integration [8], it is much less helpful in terms of practically oriented advice. When practical advice is found, it is often structured as a step-based linear model. However, in our view such linear models are vulnerable to the broader weaknesses of linear planning strategies [101,102].

Integrating NPs into primary care teams is likely to be a dynamic, complex, and messy process. In real life, many elements often need to be tackled simultaneously; it might make sense to backtrack to find and fix something that was not done right in the first place, and it is impossible to draw a line between what constitutes integration and what are normal activities. This is not to say that NP integration ought to be conceived as a something to be improvised, but rather that it is a process for which the best advice may not be step-based, as is elegantly conveyed in the often-quoted words of D. D. Eisenhower, “In preparing for battle I have always found that plans are useless, but planning is indispensable.”

The theme-based processual [101,103] perspective put forward in this article can also be linked with a particular perspective of *role theory*. In opposition to the dominant functionalist view that focuses on how a role is externally defined, this paper is aligned with the enactment, interactionist perspective. From this perspective, roles are dynamic, context dependent, processual, and interactional. The analytical focus should thus be on the everyday and local processes through which roles are constructed, negotiated, learned, enacted, and performed. Such a view is incompatible with cookbook-type linear advice. In the end, what ought to be done will always be dependent on many contingent factors. We believe the five themes delineated here can provide fruitful starting points for clinical teams striving to develop effective models for integrating new roles.

### Ethics

This study has been approved by the ethics committees of the *Comité d’éthique de la recherche de l’Agence de Santé et des Services Sociaux de Montréal* and by the *Comité d’éthique de la recherché en santé de l’Université de Montréal*.
